# Driving regeneration, instead of healing, in adult mammals: the decisive role of resident macrophages through efferocytosis

**DOI:** 10.1038/s41536-021-00151-1

**Published:** 2021-08-03

**Authors:** Lise Rabiller, Virginie Robert, Adèle Arlat, Elodie Labit, Marielle Ousset, Marie Salon, Agnès Coste, Léa Da Costa-Fernandes, Paul Monsarrat, Bruno Ségui, Mireille André, Christophe Guissard, Marie-Laure Renoud, Marine Silva, Gilles Mithieux, Isabelle Raymond-Letron, Luc Pénicaud, Anne Lorsignol, Louis Casteilla, Cécile Dromard Berthézène, Béatrice Cousin

**Affiliations:** 1 STROMALab, Université de Toulouse, CNRS ERL5311, EFS, ENVT, Inserm U1031, UPS, Toulouse, France; 2 UMR 152 Pharma Dev, Université de Toulouse, IRD, UPS, Toulouse, France; 3 UMR 1037, Centre de recherche en cancérologie de Toulouse (CRCT), Université de Toulouse, INSERM, UPS, Toulouse, France; 4 Inserm U1213, Université Lyon 1, Villeurbanne, France; 5 Present Address: Department of Physiology and Cell Information Systems, McGill University, Montreal; Alan Edwards Center for Research on Pain, McGill University, Montreal, QC Canada; 6 Present Address: RESTORE Institute, UMR 1301-Inserm 5070-CNRS EFS Univ. P. Sabatier, Toulouse, France; 7 Present Address: Department of Comparative Biology and Experimental Medicine, Faculty of Veterinary Medicine, University of Calgary, Calgary, AB Canada

**Keywords:** Physiology, Cell biology, Immunology

## Abstract

Tissue repair after lesion usually leads to scar healing and thus loss of function in adult mammals. In contrast, other adult vertebrates such as amphibians have the ability to regenerate and restore tissue homeostasis after lesion. Understanding the control of the repair outcome is thus a concerning challenge for regenerative medicine. We recently developed a model of induced tissue regeneration in adult mice allowing the comparison of the early steps of regenerative and scar healing processes. By using studies of gain and loss of function, specific cell depletion approaches, and hematopoietic chimeras we demonstrate here that tissue regeneration in adult mammals depends on an early and transient peak of granulocyte producing reactive oxygen species and an efficient efferocytosis specifically by tissue-resident macrophages. These findings highlight key and early cellular pathways able to drive tissue repair towards regeneration in adult mammals.

## Introduction

Repair processes following tissue injury result either in regeneration or in scar formation. Whereas scar healing commonly leads to the loss of functional tissue and fibrous scar development, regeneration completely recapitulates the original tissue architecture and function. The understanding of the control of repair outcome is, therefore, a concerning challenge for regenerative medicine^1^.

Regeneration has mostly been studied in lower vertebrates and in newborn mammals, since adult mammals do not regenerate, and instead heal tissue damages with a scar except in specific strains such as the spiny mouse^2^ or the MRL/MPJ mouse^3^. Most of the studies dealing with tissue repair in adult mammals thus recapitulate the healing process and not regeneration. In a recently developed model of tissue lesion, relying on massive resection of the subcutaneous fat pad, we have been able to induce adipose tissue (AT) regeneration in adult mice. By using this model to compare the regenerative and scar healing processes, we demonstrated that regeneration is controlled through the generation of an early, large, and transient peak of reactive oxygen species (ROS)^4^. Because ROS act as both signaling molecules and mediators of inflammation^5^, our findings suggested a peculiar role of the inflammatory process in tissue regeneration in adult mammals as it has been demonstrated in lower vertebrates^6^.

At a cellular scale, granulocytic neutrophils and macrophages are the key players in the inflammatory process. In both lower vertebrates and mammals, no study has yet demonstrated the role of neutrophils in regeneration. In contrast, macrophages have been involved in regeneration in salamander^7^, neonatal mice in a myocardial infarction model^8^, as well as in adults in a digitip amputation context or in Acomys mice^9^. The phagocytosis of apoptotic neutrophils by macrophages via a process called efferocytosis is one of the main mechanisms contributing to the resolution of inflammation in physiological situations^10^. This overall process is partly orchestrated by pro-and anti-inflammatory cytokines as well as eicosanoids such as prostaglandins and leukotrienes that therefore contribute to control tissue repair^11^.

Recent studies have highlighted the complexity of the macrophage population, by identifying the roles of functionally distinct macrophage subsets in tissue homeostasis and tissue repair^12–15^. Indeed, bone marrow (BM) derived monocytes recruited after tissue lesion are pro-inflammatory and exhibit tissue-destructive activity, whereas tissue-resident populations are pro-resolutive and drive tissue repair^16^. Like other adult tissues, the AT hosts distinct populations of macrophages displaying unique tissue distributions, transcriptional profiles, and functions and have distinct origins^17,18^. Among the origins, we have demonstrated that in adult mice, AT-macrophages belong to a specific and quantitatively significant hematopoietic process, relying on the presence of peculiar hematopoietic stem cells inside the AT^19,20^. Interestingly, disruption of endogenous AT hematopoiesis leads to inflammatory macrophages production that specifically contributes to chronic inflammation and AT dysfunction in contrast to macrophages derived from the BM^21^. These results suggest that in the AT, macrophages may promote beneficial or detrimental effects on tissue homeostasis depending on their local or medullar origin.

We thus hypothesized here that the early inflammatory phase following injury is crucial in the tissue repair outcome and we investigated the precise role of granulocytes and macrophages in tissue regeneration in adult mammals with a focus on macrophage origin. By using in vivo gain or loss of function studies, we show here that a rapid and transient peak of ROS-producing granulocytes is required for regeneration. In addition, macrophages derived from AT-hematopoiesis direct the repair process towards regeneration due to their efficient efferocytosis, in contrast to medullar macrophages that nurture inflammation and thus promote scar healing.

## Results

### Regenerative healing is characterized by an early and transient inflammation

**Fig. 1:**
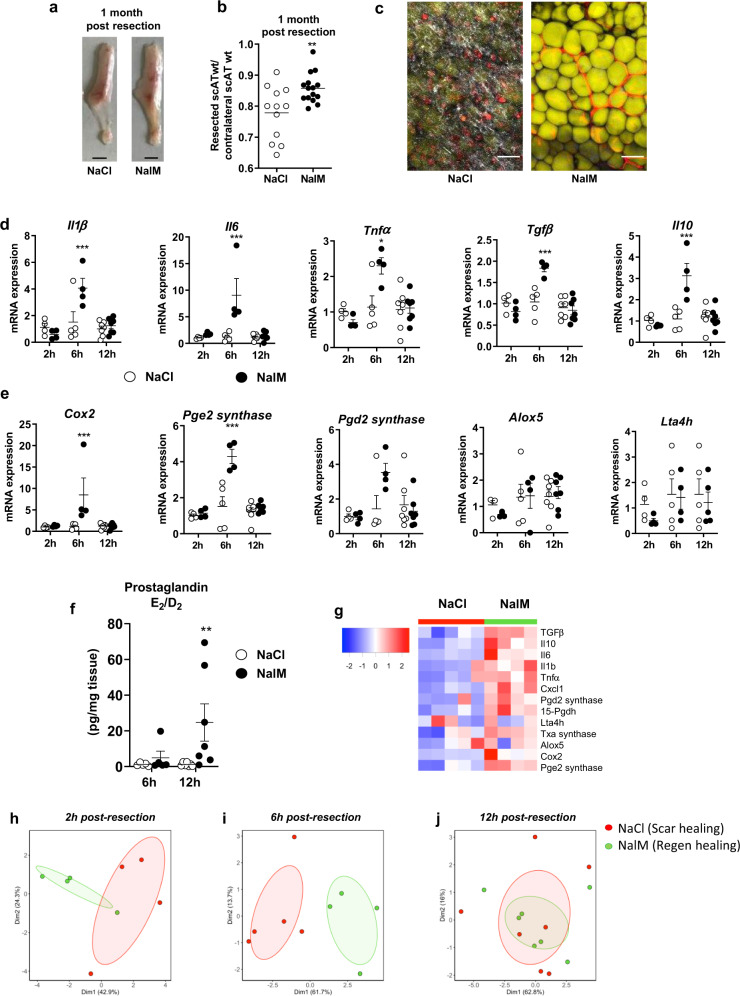
Regenerative healing is characterized by early and transient inflammation. **a** Representative pictures of scAT 1 month post-resection and NaCl (scar healing condition) or NalM (regenerative condition) treatment (scale bar: 0.5 cm). **b** Weight ratio between resected and contralateral scAT 1 month post-resection in NaCl or NalM-treated mice (*n* = 12–15 per group). **c** Representative pictures of resection plane area in NaCl and NalM treated mice one month post-resection. Adipocytes (yellow) were stained with Bodipy and vessels (red) with in vivo Lectin injection. Collagen fibers (gray) were imaged using a second harmonic generation (SHG) signal. Images were obtained using maximum intensity projections of 23 stack images. (scale bar: 50 µm). **d**, **e** Quantification by RTqPCR at 2 h, 6 h, and 12 h post-resection of mRNA encoding cytokines (Interleukin 1β, 6 (*Il1β, Il6*), Tumor Necrosis Factor α (*Tnfα*), Transforming Growth Factor β (*Tgfβ*) and interleukin 10 (*Il10*)) (**d**) and enzymes involved in lipid mediator synthesis (Cyclooxygenase 2 (*cox2)*, Prostaglandin E2 synthase (*Pge2 synthase*), Prostaglandin D2 synthase *(Pgd2 synthase)*, Arachidonate 5-lipoxygenase (*Alox5*) and Leukotriene A4 Hydrolase (*Lta4h*)) (**e**) in SVF isolated from the injured scAT of NaCl or NalM treated mice (*n* = 4–6 per group). **f** Quantification of PGD2 and PGE2 metabolites in the exudate of the resection plane of NaCl or NalM treated mice, 6 and 12 h post-resection. Results are expressed as a ratio between PGD_2_ and PGE_2_ metabolites (*n* = 5–7 per group). **g** Heatmap performed on 14 standardized gene expressions, 6 h post-resection. The dendrogram performed according to the Ward method was able to cluster between scar and regenerative healing conditions. Principal component analysis performed at 2 h (**h**) 6 h (**i**) and 12 h (**j**) post-resection on standardized gene expression. Individuals with scar healing and regenerative healing signatures were colored in red and green, respectively. Data are represented as mean ± SEM. (*p < 0.05, **p < 0.01, ***p < 0.001 between scar and regenerative healing conditions). NalM naloxone methiodide, scAT subcutaneous adipose tissue, SVF stromal vascular fraction, wt weight.

To analyze the role of the inflammatory response in the outcome of tissue lesion, we used the previously validated model of subcutaneous AT (scAT) resection^4^. Massive resection of scAT followed by treatment with NaCl or Naloxone Methiodide (NalM), an antagonist of opioid receptors, resulted in scar healing or regeneration, respectively, one month post-resection as previously described^4^. As expected and compared to NaCl (control) mice that exhibit scar healing, NalM treatment-induced macroscopic scAT regeneration (Fig. 1a) is associated with an increased weight ratio between the resected scAT and its contralateral uninjured counterpart (Fig. 1b). Regeneration was associated with the presence of unilocular fully differentiated adipocytes and new vessel formation as revealed by BODIPY and lectin staining, respectively (Fig. 1c). In contrast, scar healing was characterized by strong collagen fibers deposition as revealed by Second-Harmonic Generation imaging, and the absence of adipocyte differentiation (Fig.1c).

We first determined the temporal and dynamic inflammatory response to surgical tissue resection in both scar (NaCl) and regenerative (NalM) healing conditions. Compared to scar healing, regenerative condition was characterized by a significant and transient increase in the expression of pro-inflammatory cytokines such as interleukin (Il)1β, Il6 and tumor necrosis factor-alpha (Tnfα) as well as anti-inflammatory cytokines such as transforming growth factor-beta (Tgfβ) and Il10, 6 h post-resection, in the resection plane (Fig. 1d). This upregulation of pro- and anti-inflammatory cytokines was associated with a significant increase in expression of enzymes involved in the production of pro-inflammatory lipid mediators (Cox-2, Pge2 synthase), and to a lesser extent in Pgd2 synthase but not in the expression of enzymes involved in the leukotriene production (Alox5, Lta4h) (Fig. 1e). The induction of Cox-2 and Pge2 synthase expression was reflected by an increased ratio between prostaglandins (PG) E2 and D2 12 h post-resection (Fig. 1f). We then used heatmap visualization (Fig. 1g) and principal component analysis (PCA) (Fig. 1h-j) to determine whether regenerative and scar healing conditions can be characterized by a global gene signature. The heatmap of the expression of 12 genes (cytokines and lipid mediator synthesis enzymes) 6 h post-resection (Fig. 1g), highlighted a differential expression pattern between regenerative and scar healing conditions. PCA was then used to reduce the dimensionality of the dataset while retaining most of its original variability. Consequently, we reduced a set of correlated variables (the gene expressions) into fewer uncorrelated variables (the dimensions). The projection of the data into this new set of dimensions revealed a clear clustering between the two groups on the first and second dimensions 2 and 6 h post-resection (Fig. 1h, i), although the discrimination is clearer at 6 h than at 2 h post-resection between regenerative and scar healing conditions, as revealed by the principal components that account for 67% of the variability of the data at 2 h post-resection while it accounts for 75% at 6 h post-resection (Fig. 1h, i). Interestingly this clustering completely disappears 12 h post-resection (Fig. 1j). We thus demonstrated here that an early but transient inflammation is associated with regeneration, and that a specific pattern of gene expression 6 h post-resection may be predictive of the regenerative outcome.

### Regenerative healing depends on ROS production by neutrophils

**Fig. 2:**
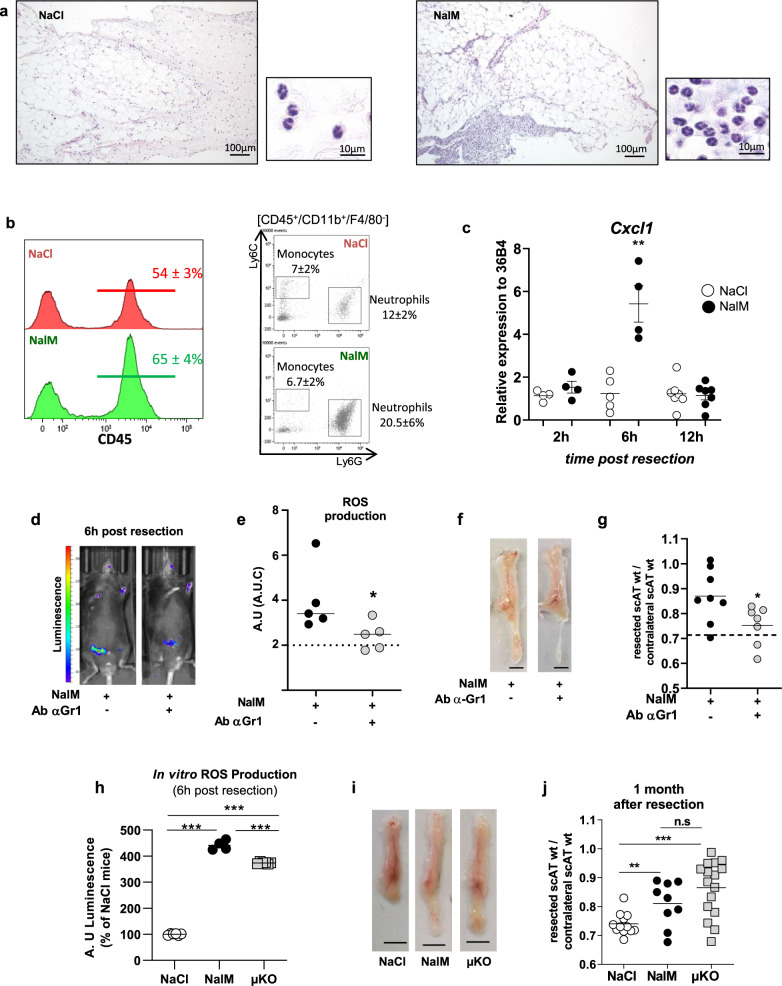
Neutrophils depletion impairs ROS production and inhibits scAT regeneration in NalM-treated mice. **a** Representative comparative microscopic aspects of the resection plane of scAT 8 h post-surgery in NaCl and NalM-treated mice (Hemalun & eosin staining). **b** Representative histograms and dot plot analyses of SVF cells isolated from NaCl (red curve) or NalM (green curve) treated mice 6 h post-resection, showing the percentage of CD45^+^ cells and granulocytes (neutrophils: CD45^+^/Ly6G^+^/Ly6C^−^/CD11b^+^ and monocytes CD45^+^/Ly6G^−^/Ly6C^+^/CD11b^+^) in the resection plane. **c** Gene expression quantification by RT-qPCR of the chemokine *Cxcl1* in SVF isolated from the resection plane of NaCl-treated or NalM-treated mice 2, 6, and 12 h post-resection (*n* = 4 per group). **d** Representative in vivo imaging of ROS production 6 h post-resection in NalM-treated mice, treated or not with anti-Gr1 blocking antibody (Ab α-Gr1). **e** Quantification of ROS production in vivo from 0 to 72 h post-resection in NalM-treated mice, treated or not with Ab α-Gr1. The dotted lines represent ROS production obtained in NaCl-treated mice (*n* = 5 per group). **f** Representative pictures of scAT 1 month post-resection, in NalM-treated mice treated or not with Ab α-Gr1 (scale bar: 0.5 cm). **g** Weight ratio between resected and contralateral scAT 1 month post-resection in NalM treated mice, treated with isotype or Ab α-Gr1 (*n* = 7–8 per group). The dotted line show values obtained in scar healing (NaCl) conditions. **h** In vitro quantification of ROS production by Gr1^+^ populations sorted from scAT of NaCl, NalM, or µKO mice 6 h post-resection (*n* = 4-6 per group). **i** Representative pictures of scAT 1 month post-resection, in NaCl or NalM-treated mice and in NaCl-treated mice knock out for the µ opioid receptor (µKO) (scale bar: 0.5 cm). **j** Weight ratio between resected and contralateral scAT 1 month post-resection in NaCl, NalM-treated and µKO mice (*n* = 9–16 per group). Data are represented as mean ± SEM (*p < 0.05, ***p < 0.001 between scar and regenerative healing conditions). AT adipose tissue, AU arbitrary units, AUC area under the curve, NalM naloxone methiodide, scAT subcutaneous adipose tissue, SVF stromal vascular fraction, ROS reactive oxygen species, wt weight.

The early inflammation was associated with an infiltration of the resection plane by small round leukocytes, of typical granulocytic neutrophil morphology (segmented or ringform nucleus with condensed chromatin and pale cytoplasm) (Fig. 2a, zoom). The granulocytic infiltration was observed in both the connective tissue and in the scAT (mainly as intravascular leukostasis and perivascular cuffings) in the resection plane (Fig. 2a), as demonstrated by histological evaluation. The nature of infiltrating leukocytes was confirmed by flow cytometry. Both neutrophils and monocytes were identified as CD45^+^/CD11b^+^/F4/80^−^/Ly6G^+^/Ly6C^−^ and CD45^+^/CD11b^+^/F4/80^−^/Ly6G^−^/Ly6C^+^, respectively (Fig. 2b), following the gating strategy shown in Supplementary Fig. 1a. Neutrophils, but not monocytes, increased in the resection plane 6 h post-resection in regenerative compared to scar healing conditions (Fig. 2a, b). Moreover, the expression of Cxcl1, a chemokine that in parallel to TNFα induces the infiltration of neutrophils^22^, was significantly and transiently increased in the injured scAT during regeneration (Fig. 2c).

To unravel the role of granulocytes in the NalM-induced regeneration process, their specific depletion was undertaken in vivo using an anti-Gr1 blocking antibody. We first verified that Gr-1 antibody revealed mainly monocytes and neutrophils (Supplementary Fig. 2a) and that anti-Gr1 injection induced a drastic and specific depletion in granulocyte, and especially neutrophils (Ly6G^+^) population in the scAT in vivo from 24 to at least 72 h after injection (Supplementary Fig. 2b, c). ScAT resection was thus performed 24 h after anti-Gr1 injection. ROS production quantified 6 h post-resection was significantly decreased in the injured scAT after anti-Gr1 treatment (Fig. 2d, e), and return to basal values (dotted line). This significant decrease in ROS production was associated with inhibition of regeneration quantified 4 weeks post-resection (Fig. 2f). Indeed, the weight ratio between resected and contralateral scAT returned to values observed in scar healing conditions (Fig. 2g). These data demonstrate for the first time that the ROS required for regeneration are produced by granulocytes. In addition, ROS production was quantified in vivo by using luminol that enables bioluminescence imaging of myeloperoxidase activity^23^ suggesting that ROS production post-resection depends mainly on neutrophils.

In parallel, ROS production was quantified in vitro in scAT-Gr1^+^ and Gr1^−^ cells sorted 6 h post-resection, from mice previously treated or not with NalM. In contrast to scAT-Gr1^−^ cells (data not shown), scAT-Gr1^+^ cells were able to produce ROS (Fig. 2h). In addition, when sorted from NalM-treated mice, scAT-Gr1^+^ cells exhibited a significantly higher ROS production than scAT-Gr1^+^ cells sorted from NaCl-treated mice (Fig. 2h) thus confirming the results obtained in vivo. NalM being an antagonist of µ opioid receptors, mice knock out for the µ opioid receptor (µKO mice), and therefore unable to respond to endogenous opioids through activation of this receptor subtype, were subjected to scAT resection before Gr1^+^ cell sorting from resection plane 6 h post-resection. ROS production was significantly higher in scAT-Gr1^+^ cells sorted from µKO mice compared to NaCl-treated mice but remained slightly lower than the production quantified in scAT-Gr1^+^ cells sorted from NalM treated mice (Fig. 2h). In order to confirm the involvement of µ receptors in the NalM effect, Gr1^+^ cells were treated with DAMGO, a selective µ opioid receptor agonist, that reverses the inhibitory effect of NalM. In this condition, the ROS production by Gr1^+^ cells sorted from NalM mice was inhibited (Supplementary Fig. 2d) suggesting again a direct effect of opioids on granulocytes through µ opioid receptors. Interestingly, this high ROS production is associated with a spontaneous scAT-regeneration after resection in µKO mice (Fig. 2i), the weight ratio between resected and contralateral scAT being similar in µKO and NalM-treated mice and significantly higher than in NaCl-treated mice (Fig. 2j). Altogether, these results demonstrate that through a direct effect on µ opioid receptors, NalM-induced ROS production by granulocytes determines tissue repair outcome.

### Efficient efferocytosis of neutrophils by CD11c^+^ macrophages is required for regeneration

**Fig. 3:**
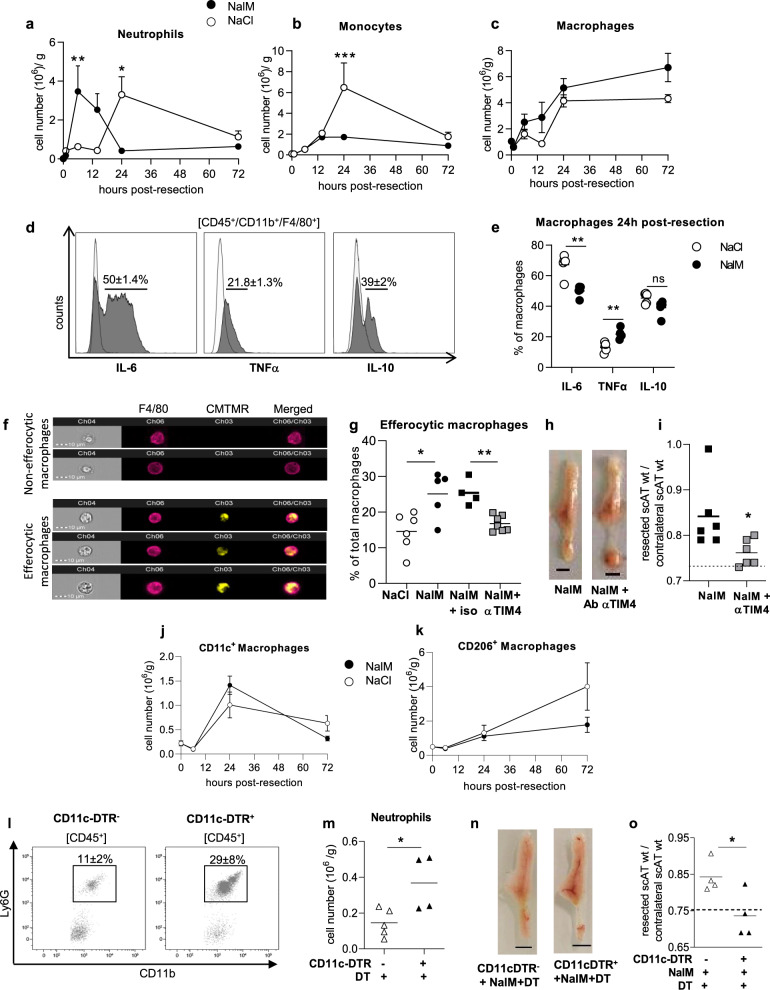
Efficient efferocytosis of apoptotic neutrophils by CD11c^+^ macrophages is required for regeneration. Time-course study of the number of neutrophils (CD45^+^/CD11b^+^/F4/80^−^/Ly6C^−^/Ly6G^+^) (**a**), monocytes (CD45^+^/CD11b^+^/F4/80^−^/Ly6C^+^/Ly6G^−^) (**b**) and macrophages (CD45^+^/CD11b^+^/F4/80^+^/Ly6G^−^) (**c**) in the scAT of NaCl and NalM treated mice (*n* = 5–13 per group). **d** Representative histogram of flow cytometry of IL-6, TNFα, and IL-10 staining (gray histogram) or isotype (white histogram) on macrophages in resection planes 24 h post-resection. **e** Percentage of IL-6, TNFα, and IL-10 producing macrophages in NaCl or NalM treated mice 24 h post-resection (*n* = 5 per group). **f** Examples of the imaging flow cytometry channels of macrophages stained with F4/80 (pink) and having engulfed (lower panels) or not (upper panels) CMTMR stained apoptotic neutrophils (yellow) in scAT of NaCl-treated mice 17 h post-resection. **g** Quantification of efferocytic macrophages in the scAT of NaCl and NalM-treated mice with isotype or TIM4 blocking antibody (*n* = 4–7 per group). **h** Representative pictures of scAT 1 month post-resection, in mice treated with NalM and with or without blocking TIM4 antibody (scale bar: 0.5 cm). **i** Weight ratio between resected and contralateral scAT 1 month post-resection in mice treated with NalM and with or without blocking TIM4 antibody (*n* = 6) The dotted line show values obtained in scar healing (NaCl) conditions. Quantification of macrophages CD11c^+^ (**j**) and CD206^+^ (**k**) in NaCl and NalM-treated mice 24 h post-resection, (*n* = 5 per group). **l** Representative dot plots of flow cytometry analyses showing neutrophils (CD45^+^/CD11b^+^/F4/80^−^/Ly6C^−^/Ly6G^+^) 24 h post-resection in scAT of CD11c-DTR^+^ and CD11c-DTR^−^ mice treated with NalM and diphtheria toxin (DT). **m** Quantification of neutrophils 24 h post-resection in CD11c-DTR^+^ and CD11c-DTR^-^ mice treated with DT and NalM (*n* = 4–5 per group). **n** Representative pictures of scAT 1 month post-resection in CD11c-DTR^+^ and CD11c-DTR^−^ mice treated with NalM and DT (scale bar: 0.5 cm). **o** Weight ratio between resected and contralateral scAT in CD11c-DTR^+^ and CD11c^-^DTR^−^ mice treated with NalM and DT (*n* = 4). The dotted line shows values obtained in scar healing (NaCl) conditions. Data are represented as mean ± SEM. (*p < 0.05, **p < 0.01 between scar and regenerative healing conditions). Ab Antibody, NalM naloxone methiodide, CMTMR 5-(and-6)-(((4-chloromethyl)benzoyl)amino)tetramethylrhodamine, scAT subcutaneous adipose tissue, wt weight.

One of the mechanisms by which granulocytes (neutrophils and monocytes) are involved in tissue repair is their transition to apoptosis followed by their clearance by macrophages (efferocytosis)^10^. We thus focused on the resolution phase of inflammation following scAT resection with or without NalM treatment, and performed a time-course study of the inflammatory cell populations during 72 h following resection, focusing on neutrophils (CD45^+^/CD11b^+^/F4/80^−^/Ly6C^−^/Ly6G^+^), monocytes (CD45^+^/CD11b^+^/F4/80^−^/Ly6C^+^/Ly6G^−^) and macrophages (CD45^+^/CD11b^+^/F4/80^+^/Ly6G^−^) following the gating strategy shown in Supplementary Fig. 1b. The resection led to an early peak (6 h) of neutrophils followed by a rapid return to basal values in the resection plane in regenerative condition, whereas the increase in neutrophils number was delayed (24 h) and persisted for 72 h post-resection in scar healing condition (Fig. 3a). The monocyte number remained low in regenerative conditions while it increased in scar healing mice 24 h post-resection and returned to basal values thereafter (Fig. 3b). The total macrophage number increased following resection, with a maximum observed at 24 h, then reaching a plateau, and no significant differences were observed between the two conditions (Fig. 3c). Most of the macrophages in the resection plane 24 h post-resection were producing IL-6 (Fig. 3d, e), and a smaller proportion was producing TNFα and IL-10 (Fig. 3d, e). The percentage of IL-6-producing macrophages was significantly lower in regenerative than in scar healing conditions, while the opposite was observed for TNFα-producing macrophages (Fig. 3e). Macrophage efferocytosis capacity was then assessed in vivo in regenerative or scar healing conditions. To this end, isolated and CMTMR-labeled neutrophils were injected in vivo 3 h before resection. Neutrophil viability was evaluated by annexin V staining. All CMTMR+ neutrophils were alive before injection, while most of them were apoptotic (Annexin V^+^) at the time of efferocytosis analysis (Supplementary Fig. 3a). Macrophages having or not engulfed neutrophils were then visualized (Fig. 3f) and quantified in the resection plane 17 h post-resection, a time-point corresponding to the progressive decrease in neutrophil number in regenerative conditions (Fig. 3a). The percentage of efferocytic macrophages was significantly higher in regenerative than in scar healing conditions (Fig. 3g). To test whether efficient efferocytosis was required for regeneration, inhibition of efferocytosis was performed in regenerative conditions by using an anti-TIM4 blocking antibody that disrupts the interaction between apoptotic cells and macrophages^24^. Isotype was used as a control. Treatment with anti-TIM4 antibody in regenerative conditions induced a drastic decrease in the percentage of efferocytic macrophages that returned to basal values (Fig. 3g), suggesting that efferocytosis was mainly mediated through TIM4 receptor in these conditions. This disruption of TIM4-mediated efferocytosis prevented NalM-induced regenerative healing, as demonstrated by both macroscopic observations (Fig. 3h) and scAT weight ratio that returned to values obtained in scar healing (NaCl) conditions (Fig. 3i). We also investigated the expression of CD36, another major receptor involved in efferocytosis. Neither the percentage of macrophages expressing CD36 nor the expression of CD36 in sorted macrophages differed between scar and regenerative healing conditions, 24 h post-resection (Supplementary Fig. 3b–d), suggesting that this receptor was not involved in the differential efferocytic activity. Altogether, these data demonstrate for the first time that an efficient TIM4-mediated efferocytosis of apoptotic neutrophils by macrophages is a crucial mechanism required for tissue regeneration.

Knowing that macrophages are a heterogeneous population differentially involved in the acute inflammatory phase of tissue repair, we then quantified both pro- and anti-inflammatory macrophages, based on the expression of CD11c and CD206 respectively. The number of pro-inflammatory (CD11c^+^) macrophages substantially increased (5×) to reach a maximum of 24 h post-resection (Fig. 3j). In contrast, the number of ant-inflammatory (CD206^+^) macrophages slightly increased (2×) from 6 to 72 h following resection (Fig. 3k). No significant difference was observed between healing and regenerative conditions.

To uncover the role of CD11c^+^ macrophages in tissue repair, we used CD11c-DTR mice, in which, CD11c^+^ cells can selectively be depleted by injection of diphtheria toxin (DT) as previously described^25^. We first confirmed that in the scAT, most of the CD11c^+^ cells expressed the macrophage markers F4/80 and CD11b (Supplementary Fig. 4a). The toxin was injected 24 h before surgery, and regeneration was analyzed 4 weeks later. As controls, we used CD11c-DTR^−^ mice injected with DT. As expected, DT treatment efficiently decreased the number of CD11c^+^ macrophages (CD45^+^/CD11b^+^/F4/80^+^) present in the resection plane 24 h post-resection (Supplementary Fig. 4b). It resulted in a significant accumulation of neutrophils (Fig. 3l, m), suggesting an altered efferocytosis. Depletion of CD11c^+^ macrophages also prevented NalM-induced tissue regeneration (Fig. 3n), and decreased the weight ratio between resected and contralateral scAT to values obtained in scar healing (NaCl) condition (Fig. 3o). These data underlined the crucial role of CD11c+ macrophages in the handling of tissue repair.

### AT resident macrophages but not classical BM-derived macrophages are required for tissue regeneration

**Fig. 4:**
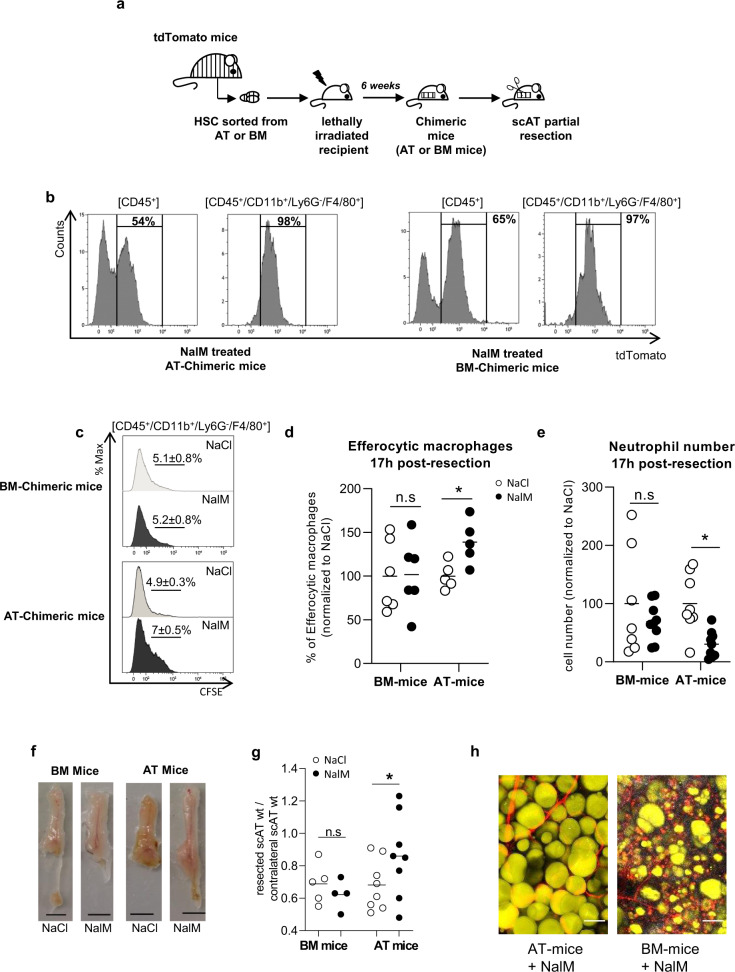
AT Resident macrophages but not classical BM-derived macrophages are required for tissue regeneration. **a** Hematopoietic chimera strategy: 2 × 10^3^ LSK cells sorted from the scAT or the bone marrow (BM) of mTmG mice were co-injected with 2 × 10^5^ total BM cells of C57Bl6 into lethally irradiated C57Bl6 recipients. Two months after hematopoietic reconstitution, scAT resection was performed and chimeric mice were treated with NaCl or NalM. **b** Representative histograms showing chimerism (tdTomato staining) in total immune cells (CD45^+^) and macrophages (CD45^+^/CD11b^+^/Ly6G^-^/F4/80^+^) in NalM-treated AT- and BM-chimeric mice, 24 h post-resection. **c** Representative histograms of CFSE staining in macrophages 24 h post-resection in NaCl and NalM-treated chimeric mice, corresponding to the percentage of macrophages having engulfed CFSE^+^ neutrophils. **d** Quantification of efferocytic macrophages 24 h post-resection in NaCl and NalM-treated chimeric mice (*n* = 5–6). **e** Quantification of neutrophil number by flow cytometry 24 h post-resection, in NaCl or NalM, treated mice (*n* = 7–8 per group). **f** Representative pictures of scAT 1 month post-resection, in BM- and AT-chimeric mice treated or not with NalM (scale bar: 0.5 cm). **g** Weight ratio between resected and contralateral scAT in AT- and BM-chimeric mice 1-month post-resection (*n* = 5–8 per group). **h** Representative pictures of resection plane area of NalM-treated AT- and BM- chimeric mice, one-month post resection. Adipocytes (yellow) were stained with Bodipy and vessels (red) with in vivo Lectin injection. Collagen fibers (gray) were imaged using second harmonic generation (SHG) signal. Images were obtained using maximum intensity projections of 23 stack images (scale bar: 50 µm). Data are represented as mean ± SEM. (*p < 0.05, **p < 0.01, ***p < 0.001 between scar and regenerative healing conditions). AT adipose tissue, BM bone marrow, LSK Lin^−^/Sca-1^+^/c^−^Kit^+^ cells, NalM naloxone methiodide, scAT subcutaneous adipose tissue, wt weight.

We previously demonstrated that scAT myeloid populations mainly originate from AT hematopoiesis rather than BM hematopoiesis in physiological conditions^20^. We thus investigated the origin of the macrophages involved in regeneration. Chimeric mice were generated by using standard repopulation assays as previously described^20^ by injecting 2 × 10^3^ scAT-LSK or BM-LSK sorted from tdTomato mice mixed with 2 × 10^5^ BM cells isolated from C57Bl/6 mice (Fig. 4a). Injection of BM cells ensured the survival of the recipient, as previously described^26^. Chimeric mice were then subjected to scAT resection 6 weeks after transplantation once the reconstitution was complete. The total chimerism and the origin of macrophages were determined by flow cytometry 24 h post-resection. Total chimerism in the AT of chimeric mice reached 50–70%, and all the macrophages present in the resection plane derived from the BM in BM-chimeric mice or from the scAT in AT-chimeric mice (Fig. 4b).

In order to compare BM and scAT-macrophage function, efferocytosis was assessed by in vivo-specific assays. Interestingly, flow cytometry analysis demonstrated a significant increase in phagocytic macrophages in the resection plane 24 h post-resection in AT- compared to BM-chimeric mice in regenerative conditions (Fig. 4c, d) associated with a significant decrease in neutrophil number (Fig. 4e) (for gating strategy, see Supplementary Fig 1b). On the contrary, in BM-chimeric mice, there was no difference in the percentage of phagocyting macrophages in vivo or in neutrophil numbers between scar and regenerative healing conditions (Fig. 4c–e). Then, to demonstrate that these differences in efferocytosis capacity drive tissue repair outcomes towards regeneration or scar healing, regeneration was assessed 4 weeks later. Macroscopic observations showed that AT-chimeric mice were able to regenerate but not BM-chimeric mice that instead displayed scar healing (Fig. 4f). In addition, we observed a significant difference in the weight ratio between resected and contralateral scAT in NalM versus NaCl treated AT-chimeric mice, while no difference was observed in BM-chimeric mice (Fig. 4g). Histological analyses of resection planes performed in NalM-treated chimeric mice revealed large unilocular fully differentiated adipocytes in AT-mice, as well as organized vascularization (Fig. 4h). In contrast resection plane in BM-mice was characterized by scattered adipocytes with small lipid droplets embedded in collagen deposition (Fig. 4h). These results suggest that macrophages play distinct role in healing process according to their origin. Altogether, these results show that macrophages originating from both the BM and the AT contribute to scar healing. However, in contrast to BM macrophages, scAT macrophages are the only ones able to exhibit efficient efferocytosis and rapid clearance of neutrophils and thus to drive tissue regeneration.

## Discussion

In this study, we compared the early cellular mechanisms driving tissue healing or regeneration in adult mammals. By using a newly developed model of tissue regeneration in a commonly used strain of mice^4^, we demonstrated that a transient and early peak of neutrophils and their ROS production, as well as their rapid elimination by resident macrophages through an efficient efferocytosis, are required for regeneration in adult mice.

Tissue injury classically leads to an early pro-inflammatory step, characterized by the production of pro-inflammatory mediators^27^. Here, the increase in the expression of pro-inflammatory cytokines in regenerative conditions shortly after scAT resection is consistent with the role of self-limiting acute inflammation in proper restorative response. Indeed, only a few studies highlight the crucial role of intense and transient production of pro-inflammatory cytokines to allow correct tissue repair^28,29^. As an example, the inhibition of IL-1β and TNFα expression by glucocorticoids leads to healing defects, and this study suggests that early induction of these genes is one of the important mechanisms in this process^28^. Among the first cytokines involved in this tissue repair, IL-6 and TNFα have been shown to promote different tissue regeneration in mammals and zebrafish^30,31^. In addition, the early and transient upregulation of IL-10 and TGFβ observed in regenerative conditions are in line with the anti-inflammatory/pro-repair activities of these cytokines^16^. Among the other signaling molecules released during inflammation are the prostaglandins. PGE2 and D2 are indeed produced in a time-dependent fashion, and actively contribute to tissue repair^32,33^. In agreement with our data, the inhibition of the PGE2-degrading enzyme, thus leading to an increase in PGE2 levels, has been shown to promote tissue repair in a mouse model of liver injury^34^. When analyzed together through PCA, our results show that the global gene expression discriminates between regenerative and scar healing conditions, but only very early after tissue lesion, suggesting that temporal cues are critical for the tissue repair outcome. This is in agreement with previous work showing that cytokines such as IL-6 are protective and limit host damage in the short term, but can also be deleterious when chronically elevated^35^. It has to be noted however, that unlike our work, most of the studies on tissue regeneration do not focus on the earliest steps of tissue repair.

The early pro-inflammatory stage following tissue injury is dominated by innate immune cells such as neutrophils and macrophages. The accumulation of granulocytes and especially neutrophils immediately after tissue damage has been largely documented^36^, and is supported by the upregulation of Cxcl1, a chemokine that contributes to attract neutrophils on the lesion site^22^. Neutrophils have been described as critical players in injury and repair although, it was unclear whether they have beneficial or detrimental healing functions^37^. In addition, ROS production was shown to either directly induce tissue damages^5^ or to be involved in regeneration process in diverse species including mammalian ones^4,38^. Here, our data clearly identify granulocytes as responsible for ROS production required for regeneration in mammals. Moreover, we demonstrated that through a direct effect on µ opioid receptor, NalM, an antagonist of this receptor induces the production of ROS by granulocytes. The µ opioid receptor has already been described on granulocytes, and in agreement with our results, it is suggested that its activation inhibits the inflammatory response^39^.

Our results highlight the involvement of macrophages in regeneration. This is in agreement with previous studies performed in different vertebrates among which Acomys or newborn mice and showing that macrophage depletion inhibits regeneration^8,9,40–42^. A bilateral link between ROS and macrophage efferocytosis has been recently proposed. Indeed, ingestion of apoptotic neutrophils by macrophages activates ROS production that in turn facilitates efferosome maturation and neutrophil degradation^43^. However, how macrophages initiate this pro-regenerative response remains unclear. Gain and loss of efferocytic function have shown that the timely clearance of apoptotic cells is required to induce healing in various mammal tissues^40,44,45^. Indeed, the prolonged presence of neutrophils in the wound triggers additional production of inflammatory mediators and is deleterious for proper tissue repair^46^. Here, we propose that this mechanism is required for regeneration. In addition, efferocytosis is mediated through TIM4, a receptor shown to be expressed in various resident macrophages including peritoneal macrophages, and required for their efferocytic activity^47^. Moreover, by using CD11c^+^ cell depletion, we identified CD11c^+^ macrophages as crucial actors in this context. It has to be noted that this strategy depletes the whole CD11c^+^ population. Although it mostly contains macrophages in these conditions, the involvement of other cell types such as dendritic cells cannot be totally excluded. In the AT, CD11c^+^ macrophages have been identified as pro-inflammatory and involved in the clearance of senescent cells^48,49^. The expression of CD11c has also been associated with both tissue resident macrophages that play a central role in non-inflammatory apoptotic cells clearance required for the maintenance of tissue homeostasis^50,51^ and monocyte-derived macrophages involved in tissue repair^52^.

Finally, one of our most striking results reveals that AT-resident macrophages derived from AT-endogenous hematopoiesis, but not BM-derived macrophages, are necessary for enabling regeneration. Indeed, using chimeric mice, we demonstrated that pro-regenerative efferocytotic macrophages originated from AT. This result emphasizes the beneficial role of myeloid cells deriving from endogenous AT hematopoiesis in tissue repair in contrast to medullar-originating cells, as already proposed in the context of cardiac remodeling after infarction^53^. In addition, these data are in line with the concept that the contribution of macrophages to tissue regeneration following injury depends on their lineage and/or developmental origin. Indeed, evidences in liver suggest that monocyte-derived macrophages can accelerate fibrosis resolution^54^ while a more recent study showed that resident dermal macrophages contribute to axon regeneration after nerve injury^55^. In the heart, resident macrophages derived from the primitive yolk sac and fetal progenitors are involved in tissue remodeling and cardiac regeneration while macrophages originating from definitive hematopoietic progenitors participate in the initiation of inflammation^56,57^.

Altogether, our findings indicate that early and transient recruitment of granulocytes producing ROS concomitant with an early inflammation is required for tissue regeneration in adult mice. We also elucidated the main role of CD11c^+^ resident macrophages in the clearance of neutrophils to promote regeneration. This work highlights a cellular pathway able to drive tissue repair towards regeneration in adult mammals and highlights potential targets that could be manipulated to induce regeneration in the context of tissue injury. Further studies characterizing the intrinsic phenotype of pro-resolutive resident macrophages will advance our understanding of their precise roles in driving tissue repair towards regeneration rather than scar formation.

## Methods

### Animals

Experiments were performed on 5–7 weeks-old male C57BL/6 mice (Envigo) and congenic male B6.129(Cg)-Gt(ROSA)26Sor^tm4(ACTB-tdTomato,-EGFP)Luo^/J (also known as mTmG mice; (Jackson Laboratories, stock No. 007676). Animals were group-housed in a controlled environment (12-h light/dark cycles at 21 °C) with unrestricted access to water and a standard chow diet in a pathogen-free animal facility. Granulocytes depletion was achieved by i.p. injection of 200 µg of anti-mouse Gr-1 blocking antibody (clone RB6-8C5, BioXCell, West Lebanon, NH, USA) 24 h before surgical AT resection. For CD11c^+^ cell depletion, transgenic CD11c-DTR mice were injected i.p. with 20 ng/kg of diphtheria toxin the day before tissue resection as previously described^21^.

Before tissue removal, mice were killed by cervical dislocation. All experiments were carried out in compliance with European Community Guidelines (2010/63/UE) and approved by the institutional ethics committee and from the Ministry of National Education, Higher Education and Research (protocol reference: 10691-201802091153445-v1).

### Sub-cutaneous AT (scAT) resection

Animals were anesthetized by inhalation of isoflurane (2.5%) and subjected to a single incision on the abdomen to access and resect 35–40% of the right scAT between lymph node and groin. The skin was then closed with three suture points. Mice were then injected subcutaneously at the surgery site, once a day from d0 to d3 post-resection with NalM (17 mg/kg, Sigma Aldrich, Saint Louis, MO, USA) or NaCl 0.9% to induce, respectively, regenerative or scar healing, as previously described^4^. ScAT regeneration was assessed by morphological observations and calculating the weight ratio between the right (resected) and the left (contralateral) fat pads.

### In vivo ROS imaging

Mice were briefly anesthetized by inhalation of isoflurane (2.5%) and injected i.p. with 5 mg of luminol (5-amnio-2,3-dihydro-1,4-Phtalazinedione, Sigma Aldrich, Saint Louis, MO, USA). In vivo bioluminescence was imaged using an IVIS Spectrum 200 (Caliper Life Science, Hopkinton, MA, USA) during 2 min exposure at different times after luminol injection. Image analyses were performed using Living Image 3.0 Software (Caliper Life Science, Hopkinton, MA, USA). The color intensity of the pictures was calibrated from 30 (min) to 330 (max). For each animal, the sham surgery area signal was subtracted to a resected area photon flux.

### Isolation of BM and adipose-derived stromal vascular cell fraction (SVF)

At necropsy, BM cells were immediately flushed from the sampled femurs with α-MEM medium (Life Technologies). Resection planes were carefully dissected, mechanically dissociated, and digested at 37 °C with collagenase (Roche Diagnostics, Mannheim, Germany) for 30 min. Cells from the scAT SVF were collected by centrifugation after elimination of undigested fragments by filtration as previously described^20^. Red blood cells were removed by incubation in hemolysis buffer (140 mM NH_4_Cl and 20 mM Tris, pH 7.6). Cells were then counted and used for flow cytometry, cell sorting in vitro ROS quantification, or Real-Time PCR.

### Competitive repopulation assays

Competitive repopulation assays were conducted as described previously^20^. Briefly, 2 × 10^3^ Lin^−^/Sca-1^+^/c-Kit^+^ (LSK) cells sorted from the scAT or the BM of donor mice were mixed with 2 × 10^5^ competitors BM total cells from C57Bl/6 mice. Donor mice were mT/mG mice expressing Tomato ubiquitously. In all the experiments, control and experimental LSK cells were sorted from animals of equal age. LSK purity was determined by flow cytometry and was between 92% and 97%. The mixed population was injected i.v. into lethally irradiated (10 Gy, ^137^Cs source) recipient mice of equal age. Mice reconstituted with AT- or BM-LSK (AT-mice or BM-mice) were then allowed to recover for 6 weeks. Chimerism was assessed by quantifying Tomato^+^ (dT^+^) cells among total CD45^+^ cells in the SVF.

### RNA extraction and real-time PCR

Total RNA was isolated from SVF by RLT:ethanol 100% extraction and purified using a Microprep kit (Zymo). 250 ng of total RNA was reverse-transcribed using the High Capacity cDNA Reverse Transcription kit (Life Technologies/Applied Biosystem), SYBR Green PCR Master Mix (Life Technologies/Applied Biosystem), and 300 nmol/L primers (Supplementary Table 1) on an Applied Biosystem StepOne instrument. All relative gene expression was determined using the ∆∆CT method and normalized to the 36B4 level.

### ELISA cytokines titration

At necropsy, exudates were collected at the resection plane 6 and 12 h post-resection (400 µL). The release of PGD2 and PGE2 was determined with a commercially available OptiEIA kit (BD Biosciences) according to the manufacturer’s instructions.

### Flow cytometry analysis and cell sorting

Freshly isolated SVF cells were stained in PBS containing FcR-blocking reagent for 30 min on ice with fluorochrome-conjugated antibodies. Phenotyping was performed by immunostaining with conjugated rat anti-mouse Abs and compared with isotype-matched control Abs (Supplementary Table 2). Cell apoptosis was assessed by AnnexinV (eBioscience) and DAPI staining according to the manufacturer’s instructions. Cells were then analyzed on an LSR Fortessa flow cytometer (BD Biosciences). Data acquisition and analysis were performed using Diva (Becton Dickinson) and Kalusa version 1.2 (Beckman Coulter) software, respectively, following gating strategies indicated in Supplementary Fig 1.

For LSK cell-sorting experiments, SVF cells were stained with Ly-6A/E (Sca-1), CD117 (c-Kit) antibodies, and Lineage Panel (Lin). Cells negative for lineage markers were gated, and Sca-1 and CD117 double-positive cells were sorted (BD FACSAria Fusion III Cell sorter, BD Biosciences). Enrichment of the LSK was determined by flow cytometry and varied between 92% and 97%. A similar strategy was used to sort macrophages on the basis of CD45, CD11b, and F4/80 expression (Supplementary Table 2).

### In vivo efferocytosis assay

Neutrophils from C57Bl/6 mice were isolated from the BM using a neutrophil isolation kit according to the manufacturer’s instructions (Miltenyi). Neutrophils were then stained with 2.5 µM of CellTrace carboxyfluorescein succinimidyl ester (CFSE; Molecular Probe) or 5 µM of chloromethyl-benzoyl-aminotetramethyl-rhodamine (CMTMR; Molecular Probe). Three hours before scAT resection, 3 × 10^6^ neutrophils were injected i.v. into wild-type, AT- or BM-chimeric mice. The resection plane was removed 17 h after resection and the proportion of macrophages CFSE^+^ or CMTMR^+^ was visualized and quantified by Image flow cytometry and flow cytometry, respectively, as described previously^58^.

Efferocytosis inhibition was achieved by three successive i.v. injections of anti-mouse TIM4 blocking antibody (TIM4 Ab; 0.5 mg/mice/injection) (BioXCell, West Lebanon, NH, USA) or its isotype control (rat IgG2b clone LTF-2, BioXCell) 12 h before and 3 and 17 h after surgery.

### Image flow cytometry

Individual cell images were acquired using IDEAS software (Amnis Merck Millipore, Billerica, MA, USA) on a 3-laser 6-channel imaging flow cytometer (Image Stream X Mark II, Amnis Merck Millipore) with ×40 magnification. For each data file, at least 50,000 single cells were acquired—debris and doublets were excluded based on their area and aspect ratio. Single-stain controls were acquired (all channels on, no brightfield and no side scatter image), a compensation matrix was calculated and then applied to the data files using IDEAS software (Amnis Merck Millipore). Briefly, focus cells were identified using the gradient RMS feature of the brightfield channel (Ch04). Single cells were then identified from debris and cell clusters using a plot of aspect ratio vs. area of the brightfield channel. Finally, we analyzed single cells with plot based on the intensity of CMTMR and F4/80 staining (Ch03 and CH06, respectively).

### Histological evaluation

3 h after surgical resection, resection planes were sampled at necropsy, fixed in 10% neutral buffered formalin and paraffin-embedded. Three µm-thick paraffin sections were stained with hematoxylin and eosin and microscopically evaluated by a pathologist.

### Biphoton microscopy

The mice were injected in vivo with retro-orbital TRITC- Griffonia Simplicifolia Lectin (Eurobio) to achieve proper vessel labeling. At 30 min after in vivo lectin injection, animals were euthanized and scAT was removed, oriented, and fixed overnight. scAT was embedded in 2% agarose gel and cut into 300 μm slices, then incubated in Bodipy in PBS/0.2% triton 1 h at room temperature. Imaging was performed using a Biphotonic Laser Scanning microscope (LSM880—Carl Zeiss, Jena, Germany) with an objective lens LCI’Plan Apochromat’ ×10/0.45 and excited using 488 nm(bodipy), 561 nm(lectin), and 840 nm (SHG) lasers. Images obtained were treated with Fiji Software.

### In vitro ROS quantification

The oxygen-dependent respiratory production was measured by chemiluminescence in the presence of luminol (66 μM, Sigma-Aldrich) using a thermostatically monitored luminometer (37 °C) (210410A EnVision Multilabel Reader) on 14 × 10^4^ Gr1^−^ or Gr1^+^ cells sorted from SVF. DAMGO ([D-Ala2, N-Me-Phe4, Gly5-ol]-Enkephalin acetate salt, Sigma Aldrich) was added 5 min before luminescence measure. The luminol detects both reactive oxygen and nitrogen intermediates (O2.–, ONOO–, OH.). Chemiluminescence was continuously monitored for 1 h. The ROS level was quantified and compared between different conditions using the area under the curve.

### Statistical analyses

Experiments done previously were used to determine animal sample size with adequate statistical power. The number of animals used in each study is indicated in the figure legends. Mice were randomized allocated to the different groups and investigators were blinded to analyses. All results are given as means ± SEM. Normality was checked using the D’Agostino and Pearson omnibus normality test, and variance between groups was compared. Statistical differences were measured using an unpaired two-sided Student’s *t*-test, or a nonparametric test (Mann–Whitney) when data did not pass the normality test, or when variances between groups were different. Data were analyzed using an ANOVA test when there were more than two groups. All statistical analyses were carried out using GraphPad Prism 9.0 software and a two-tailed *p* value with a 95% confidence interval was acquired. *p* < 0.05 was considered as significant. The following symbols for statistical significance were used throughout the manuscript: *p < 0.05; **p < 0.01; ***p < 0.001.

For gene expression at 6 and 12 h, a principal component analysis (PCA) was also performed as an unsupervised dimension reduction method. R software 3.6.3 with the “factoextra” 1.0.6 and “FactoMineR” 2.3 packages were used.

### Reporting summary

Further information on research design is available in the Nature Research Reporting Summary linked to this article.

## Supplementary information

Supplementary figures and tables

Reporting Summary

## Data Availability

The data that support the findings of this study are available from the corresponding author upon reasonable request.
