# Supplementary material for: Driving regeneration, instead of healing, in adult mammals: the decisive role of resident macrophages through efferocytosis
Source: NPJ Regen Med. 2021 Aug 3;6:41. doi: 10.1038/s41536-021-00151-1 (PMC8333253; doi:10.1038/s41536-021-00151-1)
Supplement: Supplementary file 1 — Supplementary figures and tables [file 41536_2021_151_MOESM1_ESM.pdf]

Supplementary Fig 1

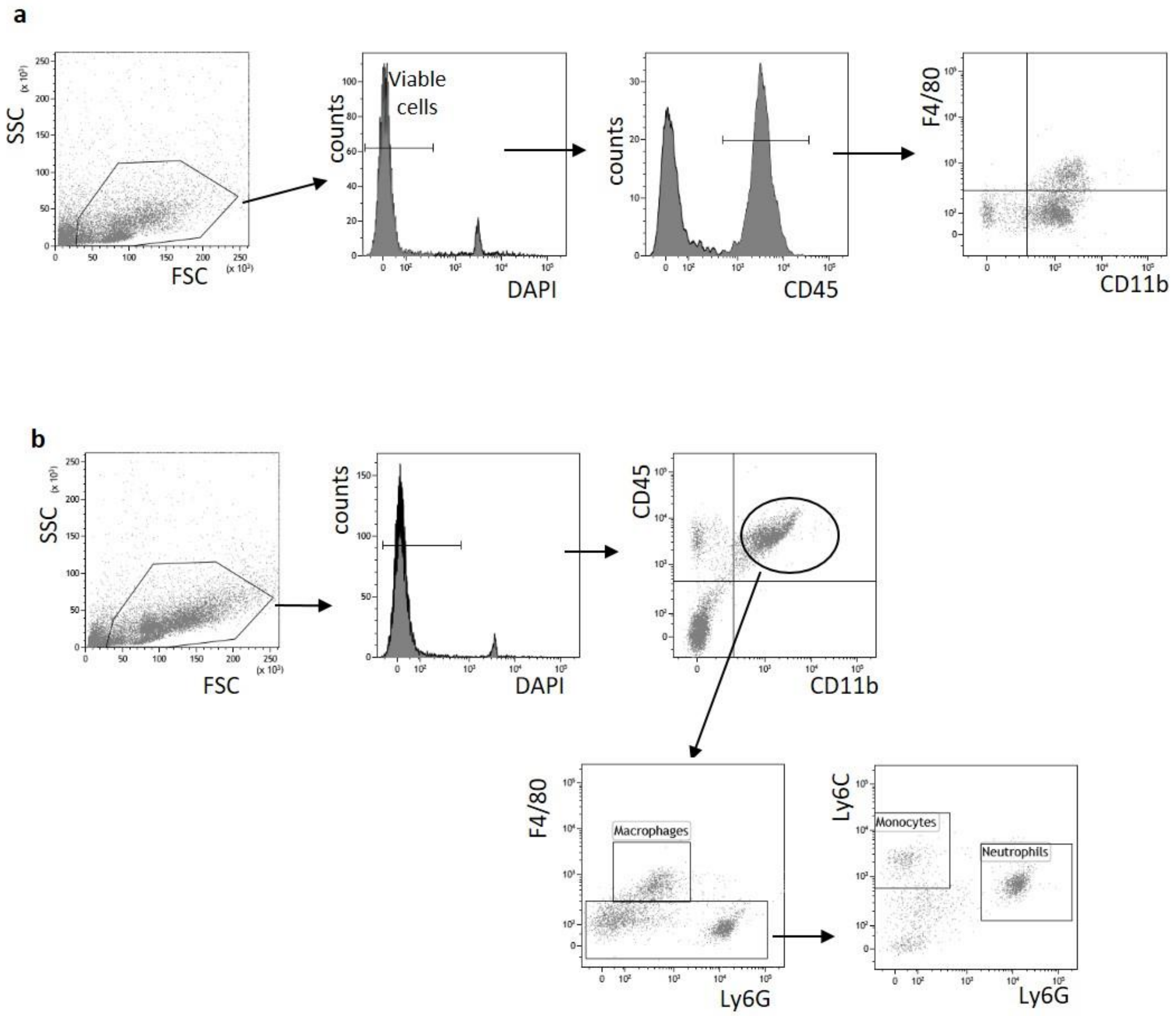

Supplementary Figure 1: Hierarchical gating strategies for flow cytometry analyses.

(a) Cells were first gated on FSC/SSC to eliminate debris, before gating on DAPI- to identify viable cells. F4/80 and CD11b staining was then analyzed on CD45+ cells. To accurately define positive versus negative cells for each antibody, isotype controls were used. **(b)** Cells were first gated on FSC/SSC to eliminate debris, before gating on DAPI- to identify viable cells. CD45+/CD11b+ cells were then analyzed to identify macrophages, neutrophils and monocytes, according to the expression of F4/80, Ly6C and Ly6G. Isotype controls were used to define positive versus negative subsets. This gating strategy was applied in Figures 3 and 4.

Supplementary Fig 2

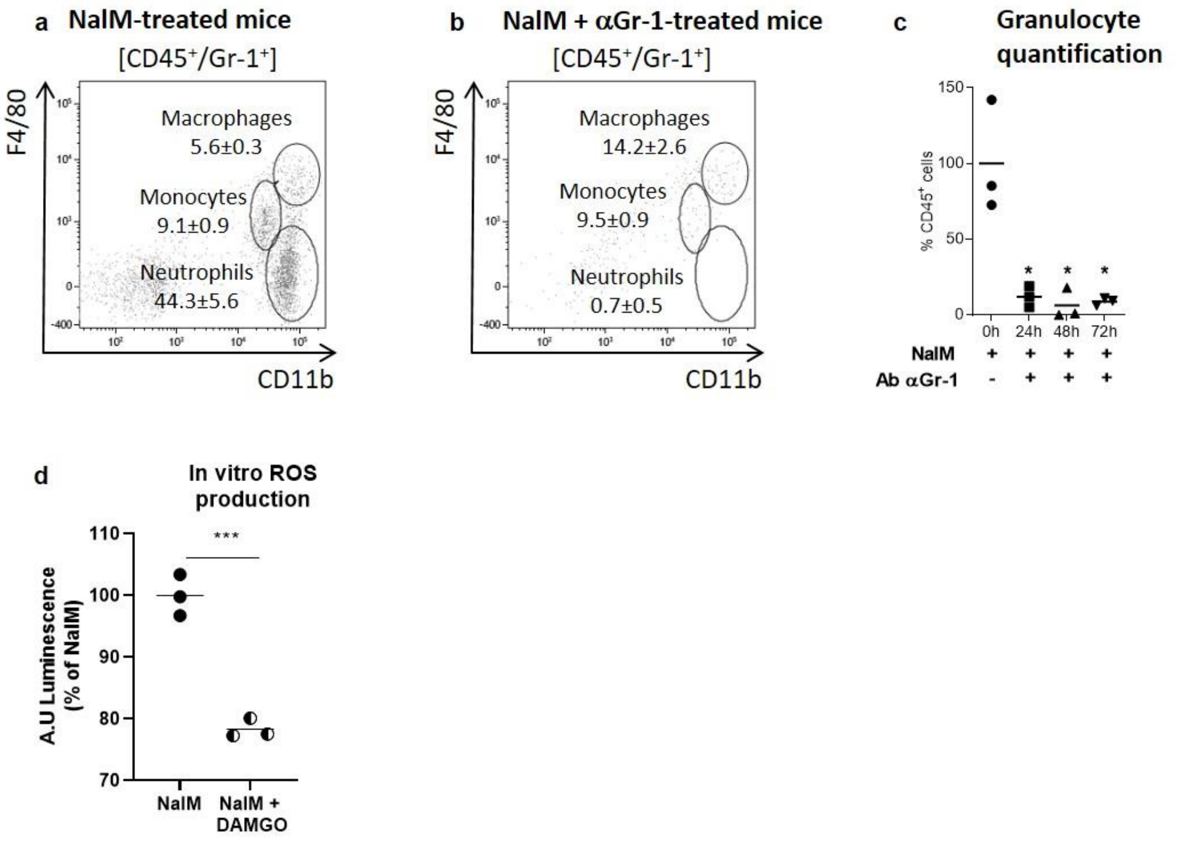

Supplementary Figure 2: In vivo treatment with anti-Gr-1 antibody depletes neutrophil population.

(a, b) Representative cytometry analyses of SVF cells isolated from NalM (A) or NalM + anti-Gr1 blocking antibody (Ab α-Gr1) **(b)** treated mice 72h post-injection, showing CD11b and F4/80 staining in $CD45^+$/ $Gr1^+$ population in the front lesion area. **(c)** Quantification of $CD45^+$/$Gr1^+$ cells in scAT 24, 48 and 72h after treatment with or without Ab α-Gr1 in NalM treated mice (n=3). **(d)** In vitro quantification of ROS production by $Gr1^+$ populations sorted from scAT of NalM mice treated or not with the selective μ-opioid receptor agonist DAMGO, 6h post-resection (n=3 per group). Data are represented as mean ± SEM. (*$p < 0.05$; ***$p<0.001$). Ab: Antibody, NalM: Naloxone Methiodide, SVF: Stromal Vascular Fraction, DAMGO: [D-Ala 2, N-MePhe 4, Gly-ol]-enkephalin.

Supplementary Fig 3

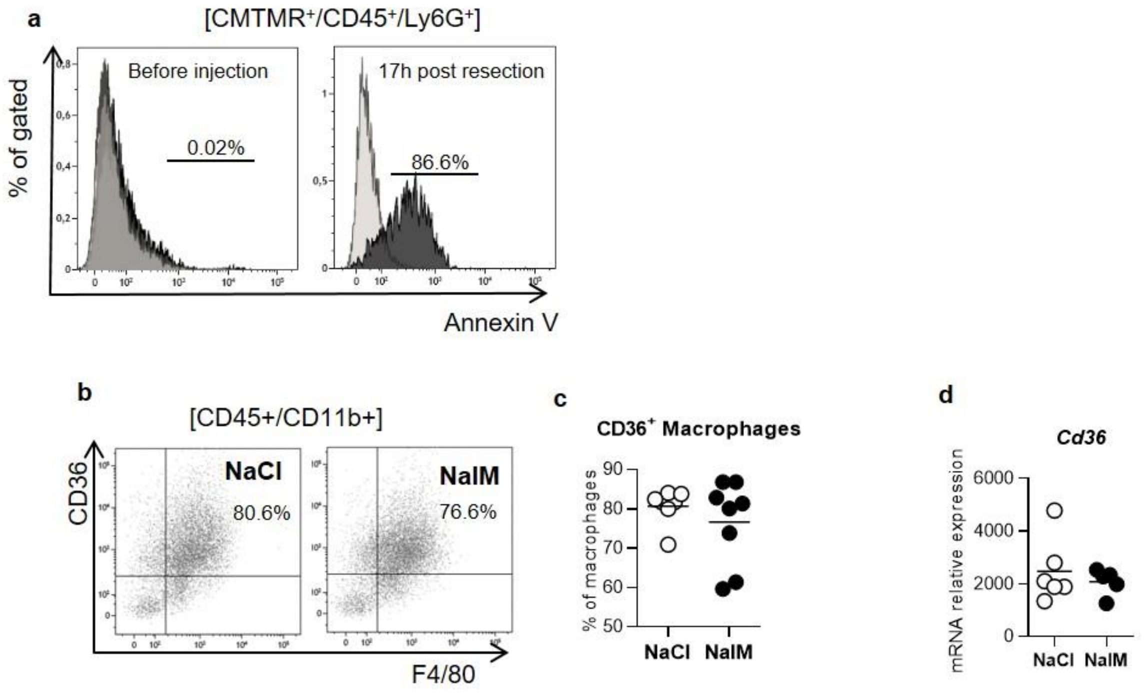

Supplementary Figure 3: Neutrophil apoptosis and CD36 expression in macrophages.

(a) Representative flow cytometry histograms showing the percentage of annexin V+ cells in the $CD45^+$/$Ly6G^+$/$CMTMR^+$ population before injection (left) and 17h post-resection (right). White histograms represent isotype controls. **(b)** Representative dot plots of CD36 staining on macrophages 24h post-resection in scar (NaCl) and regenerative (NalM) healing conditions. **(c)** Quantification of $CD36^+$ macrophages 24h post-resection in scar (NaCl) and regenerative (NalM) healing conditions (n=7-8 per group). **(d)** Quantification by RT-qPCR at 24h post-resection of mRNA encoding cd36 in macrophages sorted from the resection plane of scar (NaCl) or regenerative (NalM) conditions (n=5 per group). Data are represented as mean ± SEM. NalM: Naloxone Methiodide, scAT: subcutaneous Adipose Tissue.

Supplementary Fig 4

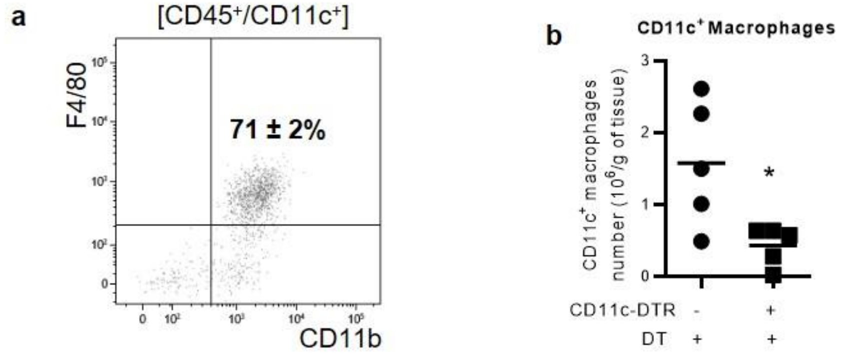

Supplementary Figure 4: In vivo treatment with diphtheria toxin depletes CD11c$^+$ macrophages in scAT.

(a) Representative flow cytometry analysis of CD11b and F4/80 staining in the CD45$^+$/CD11c$^+$ population. **(b)** Quantification of CD11c$^+$ macrophages 24h post-resection in CD11c-DTR$^+$ and CD11c-DTR$^-$ mice treated with DT and NalM (n= 5 per group). Data are represented as mean ± SEM. DT: Diphteria Toxin, NalM: Naloxone Methiodide, scAT: subcutaneous Adipose Tissue.

Supplementary Table 1: Murine primer sequences used in qPCR analyses

Gene	Forward primer	Reverse primer
36b4	AGTCGGAGGAATCAGATGAGGAT	GGCTGACTTGGTTGCTTTGG
Pge2 synthase	ATGAGGCTGCGGAAGAAGG	GCCGAGGAAGAGGAAAGGATAG
Cox2	AACCGAGTCGTTCTGCCAAT	CTAGGGAGGGGACTGCTCAT
Alox5	GTGCTGCTTGAGGATGTGAA	CTACGATGTCACCGTGGATG
Pgd2 synthase	TGGGAAGACAGCGTTGGAG	AGGCGAGGTGCTTGATGTG
Lta4h	CGTGCCCTTAGTTCCACATT	GTTGACAGCTGAACCCCAGT
Il6	TTCCTCTCTGCAAGAGACTTC	CTGCAAGTGCATCATCGTTGT
Il1β	TGCCACCTTTTGACAGTGATG	TTGGAAGCAGCCCTTCATCTT
Tnfα	GGCGGTGCCTATGTCTCA	CGATCACCCCGAAGTTCAGTA
Tgfβ	TGATACGCCTGAGTGGCTGTCT	CACAAGAGCAGTGAGCGCTGAA
Il10	CGGGAAGACAATAACTGCACCC	CGGTTAGCAGTATGTTGTCCAGC
Cxcl1	CCGAAGTCATAGCCACACTCA	CTCCGTTACTTGGGGACACC
cd36	GATGTGGAACCCATAACTGGATTCAC	GGTCCCAGTCTCATTTAGCCACAGTA

Supplementary Table 2: Anti-mouse antobodies used in flow cytometry analyses

Antibody	Clone	Manufacter
**CD45**	30F11	BD Bioscience
**CD117**	2B8	BD Bioscience
**CD11b**	M1/70	BD Bioscience
**CD11c**	N418	eBiosciences
**CD206**	MR6F3	eBiosciences
**F4/80**	BM8	eBiosciences
**Lin**	145-2C11	BD Bioscience
**Ly6G**	1A8	BD Bioscience
**Ly6C**	AL-21	BD Biosciences
**Gr-1**	RB6-8C5	BD Biosciences
**Sca1**	D7	eBiosciences
**IL6**	MP5-20F3	BD Biosciences
**IL10**	JES5-16E3	BD Biosciences
**TNFα**	MP6-XT22	eBiosciences
